# Do TSH, FT3, and FT4 Impact BAT Visualization of Clinical FDG-PET/CT Images?

**DOI:** 10.1155/2018/4898365

**Published:** 2018-02-13

**Authors:** Ryuichi Nishii, Shigeki Nagamachi, Youichi Mizutani, Tamasa Terada, Syogo Kiyohara, Hideyuki Wakamatsu, Seigo Fujita, Tatsuya Higashi, Keiichiro Yoshinaga, Tsuneo Saga, Toshinori Hirai

**Affiliations:** ^1^Department of Molecular Imaging and Theranostics, National Institute of Radiological Sciences (NIRS), QST, 4-9-1 Anagawa, Inage-ku, Chiba-shi, Chiba 263-8555, Japan; ^2^Department of Radiology, Faculty of Medicine, University of Miyazaki, 5200 Kihara, Kiyotake-cho, Miyazaki-shi, Miyazaki 889-1692, Japan; ^3^Department of Radiology, Faculty of Medicine, Fukuoka University, 7-45-1 Nanakuma, Jonan-ku, Fukuoka 814-0180, Japan; ^4^Department of Diagnostic Imaging and Nuclear Medicine, Kyoto University, Graduate School of Medicine, No. 54, ShogoinKawahara-cho, Sakyo-ku, Kyoto, Kyoto 606-8507, Japan

## Abstract

**Objective:**

We retrospectively analyzed activated BAT visualization on FDG-PET/CT in patients with various conditions and TH levels to clarify the relationships between visualization of BAT on FDG-PET/CT and the effect of TH.

**Methods:**

Patients who underwent clinical FDG-PET/CT were reviewed and we categorized patients into 5 groups: (i) thyroid hormone withdrawal (THW) group; (ii) recombinant human thyrotropin (rhTSH) group; (iii) hypothyroidism group; (iv) hyperthyroidism group; and (v) BAT group. A total of sixty-two FDG-PET/CT imaging studies in fifty-nine patients were performed. To compare each group, gender; age; body weight; serum TSH, FT3, and FT4 levels; and outside temperature were evaluated.

**Results:**

No significant visualization of BAT was noted in any of the images in the THW, rhTSH, hypothyroidism, and hyperthyroidism groups. All patients in the BAT group were in a euthyroid state. When the BAT-negative and BAT-positive patient groups were compared, it was noted that the minimum and maximum temperature on the day of the PET study and maximum temperature of the one day before the PET study were significantly lower in BAT-positive group than in all those of other groups.

**Conclusions:**

Elevated TSH condition before RIT, hyperthyroidism, or hypothyroidism did not significantly impact BAT visualization of clinical FDG-PET/CT images.

## 1. Introduction

Detection of ^18^F-2-fluoro-2-deoxy-d-glucose (FDG) uptake on positron emission tomography (PET) is commonly used for the diagnostic imaging of a variety of tumors. Currently, FDG-PET or FDG-PET/CT is the first-choice modality for functional imaging in clinical oncology [1]. However, the visualization of hypermetabolic brown adipose tissue (BAT) on FDG-PET/CT is a known cause of both false-positive and false-negative findings. Therefore, the high FDG avidity of BAT should be taken into account on FDG-PET/CT for oncologic imaging because misidentifying BAT as cancer metastasis may alter clinical decisions.

There are significant BAT depots from the anterior neck to the thorax of adults, not just in that of newborns and young children [2]. Uncoupling protein 1 (UCP1) has been identified as a biological landmark of BAT that mediates nonshivering thermogenesis. Jeanguillaume et al. reported a relationship between activated BAT on FDG-PET and UCP1 expression in mice [3]. In addition, type 2 idothyronine deiodinase (D2) is the primary enzyme responsible for the rapid increases in intracellular T3 and preservation of T3 as serum T4 decreases, which is essential for adaptive thermogenesis of BAT [4]. D2 is considered to be important for the synergism of thyroid hormone (TH) and is required to generate T3 from T4 in BAT, which maintains normal acute thermogenic function [5]. Since possible stimulation of thermogenesis in BAT by thyroid-stimulating hormone (TSH) was described in 1975 [6], several studies have confirmed the relationship between TSH or TH and the activation of thermogenesis in BAT [4, 5, 7]. However, relatively few papers have reported the relationship between TH and BAT visualization by clinical FDG-PET/CT. Lahesmaa et al. found that hyperthyroidism promotes BAT metabolism in humans using FDG-PET/CT [8]; however, contradicting results were reported by Connolly et al. in 2015 [9]. Furthermore, a case report of MRI and infrared thermal imaging regarding the presence of BAT in an adolescent with primary hypothyroidism was recently published [9]. Nonetheless, to date, details about the relationship between visualization of BAT on FDG-PET/CT and the effect of TH remain unknown. Therefore, identification of a possible mechanism of TH-induced BAT activation is considered to be important to interpret FDG-PET/CT imaging, especially of the neck region.

This study is the first to retrospectively analyze BAT visualization on FDG-PET/CT in patients with various conditions and TH levels.

## 2. Materials and Methods

### 2.1. Patients

We retrospectively reviewed images from all patients who underwent clinical FDG-PET/CT studies in our facility from October 2010 to July 2015. Patients younger than 20 years were excluded from this study. All patients who met the criteria for inclusion in any of the following five groups were enrolled in this study: (i) a TH withdrawal (THW) group, which included patients who were examined by FDG-PET/CT during a hypothyroid state with TH withdrawal and low-iodine diet prior to radioiodine therapy for metastasis after total thyroidectomy. A hypothyroid state was confirmed by serum TSH, FT3, and FT4 measured within 3 days of FDG-PET/CT; (ii) a recombinant human thyrotropin (rhTSH) group, which included patients who were examined by FDG-PET/CT after two consecutive daily intramuscular injections of rhTSH (0.9 mg of Thyrogen; Genzyme Co., Cambridge, MA) at 48 and 24 h prior to the FDG-PET/CT study, during low-iodine diet prior to radioiodine thyroid remnant ablation after total thyroidectomy. FT3, FT4, and elevated TSH levels were confirmed by blood tests on the day of FDG-PET/CT imaging; (iii) a hypothyroidism group; (iv) a hyperthyroidism group, which included patients who received treatment for hypothyroidism and were examined by FDG-PET/CT for detection of malignancy. A hypothyroid state was confirmed by an endocrinologist and serum TSH, FT3, and FT4 measured within 14 days of FDG-PET/CT; and (v) a BAT group, which included patients who were examined by FDG-PET/CT for the detection of malignancy with findings of BAT uptake and serum TSH, FT3, and FT4 levels measured within 3 months of FDG-PET/CT imaging.

This retrospective investigation was approved by institutional review board and all enrolled patients provided written informed consent that was approved by our institutional review board.

### 2.2. ^18^F-FDG-PET/CT Imaging

Commercially available FDG (185 MBq) in 2 ml of saline solution was purchased from Nihon Medi-phyisics Co. Ltd. (Tokyo, Japan). All patients were examined by whole-body PET with an integrated 16-slice multidetector CT (Siemens Biograph-16 PET/CT, Nashville, TN, USA).

The patients fasted for more than 5 h before receiving an intravenous injection of FDG (185 MBq). Whole-body PET/CT images (head to upper thigh) were acquired after 60 min for each patient in using 5 or 6 bed positions, depending on subject's height. Emission images were acquired for 2 min per bed position. The data was reconstructed using the ordered subsets expectation maximization (OSEM) method using eight subsets, two iterations, and an array size of 256 × 256. For the attenuation correction of PET/CT fusion images, the CT component was performed according to a standard protocol with the following parameters: 140 kV; 50 mAs; tube rotation time, 0.5 s per rotation; slice thickness, 5 mm; and gap, 2 mm. The E-soft workstation (Siemens, Nashville, TN, USA) was used to construct PET/CT fusion images.

### 2.3. Data Analysis

The parameters of gender; age; body weight; serum TSH, FT3, and FT4 levels; minimum and maximum temperature on the day of FDG-PET/CT imaging and maximum temperature one day before; and the deference of temperature were compared. Based on an in-house reference value, the normal range of the value of each blood hormone was set as follows: TSH, 0.35–4.94 *μ*IU/mL; FT3, 1.71–3.71 pg/mL; and FT4, 0.70–1.48 ng/dL. Temperature obtained from local weather records was referenced. Differences in temperature were calculated as follows: (maximum temperature one day before) − (minimum temperature on the day of examination) (°C).

PET/CT images were interpreted and visually assessed by one or two of the five experienced nuclear medicine physicians (SN, RN, YM, SK, and HW) with access to all available clinical information to determine whether visualization of activated BAT was apparent in the neck, shoulder, back, paravertebral, and/or other soft tissues. A final diagnosis was made by consensus.

### 2.4. Statistical Analysis

All values are expressed as the mean ± standard deviation. All statistical analyses were performed using JMP ver. 12 statistical software (SAS Institute, Cary, NC, USA). A probability (*p*) value < 0.01 was considered statistically significant. Comparisons between groups were made using the Wilcoxon rank-sum test.

## 3. Results

As summarized in Table 1, 62 FDG-PET/CT images of 59 patients were reviewed in this retrospective study, as follows: (i) THW group: 21 images; mean age, 61.6 ± 14.9 years; seven males and 14 females; (ii) rhTSH group: eight images; mean age, 48.4 ± 14.1 years; four males and four females; (iii) hypothyroidism group: nine images; mean age, 65.8 ± 17.5 years; four males and five females; (iv) hyperthyroidism group: 14 images; mean age, 50.0 ± 13.3 years; six males and eight females; and (v) BAT group: 10 images; mean age 52.5 ± 22.9 years; two males and eight females. A total of 9,273 whole-body FDG-PET/CT imaging study was performed in this study period. Among them, BAT visualization was found in 85 FDG-PET/CT imaging studies (0.92%). Among them 10 out of these 85 imaging studies whose serum TSH, FT3, and FT4 levels were able to be measured were analyzed in this study.

**Figure 1:**
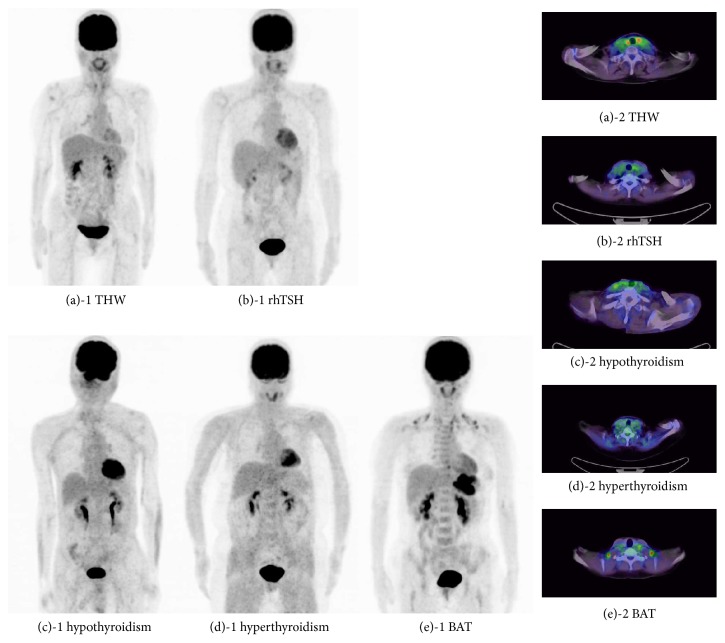
Representative whole-body maximum intensity projection and FDG-PET/CT axial images of various conditions associated with TH. Images (a) to (d) were without any uptake of BAT, but (e) showed BAT uptake in the neck, the shoulder, the back, and the paravertebral region. (a) Forty-nine-year-old female who was in THW state before radioiodine therapy (case #(4)); (b) fifty-year-old female who received rhTSH before thyroidal ablation (case #(26)); (c) seventy-three-year-old male who underwent FDG-PET for screening of recurrence after surgery for hypopharyngeal carcinoma (case #(37)); (d) fifty-two-year-old female who had a diagnosis of hyperthyroidism (case #(45)); and (e) forty-one-year-old female who received FDG-PET for gastric cancer (case #(56)).

No significant visualization of BAT was noted in any of the images included in the THW, rhTSH, hypothyroidism, and hyperthyroidism groups (Table 1 and Figure 1). Serum TSH level was significantly high in the rhTSH group and significantly low in the hyperthyroidism group. Elevated serum TSH was noted in the THW and hypothyroidism groups. All patients in the BAT group were in a euthyroid state (Table 2). Serum total cholesterol was significantly elevated in the THW group because of hypometabolism caused by hypothyroidism due to TH withdrawal.

Comparisons of BAT-negative and BAT-positive patients are shown in Table 3. The minimum and maximum temperature on the day of the PET study and maximum temperature one day before were significantly lower in BAT-positive patients, whereas there was no difference between maximum temperature one day before and minimum temperature on the day of the PET study.

Representative whole-body maximum intensity projection and FDG-PET/CT axial images of various conditions associated with TH are shown in Figures 1(a)–1(d). A representative case of visualized BAT on a FDG-PET/CT image is shown in Figure 1(e). Patients (a), (b), and (c) had high serum TSH levels at the time of FDG-PET/CT examinations, whereas patient (d) had suppressed serum TSH and patient (e) exhibited a euthyroid state. As FDG-PET/CT imaging of patient (e) was performed to identify the primary tumor site and, for the staging of gastric cancer, a focal increased uptake of FDG was detected in the left upper abdomen showing the primary site of the gastric cancer. Multiple foci of FDG uptake by BAT were seen in the neck, shoulder, back, and paravertebral regions. However, no abnormal uptake of FDG by a suspected metastatic lesion was detected.

There was also no significant higher glucose uptake in the supraclavicular subcutaneous fat and/nor in the skeletal muscle in the hyperthyroidism group compared with the BAT group, as shown in the supplement file.

## 4. Discussion

TH plays a key role in the regulation of basal metabolic rate and adaptive thermogenesis, both of which have significant impacts on body weight [10]. Adrenergic stimulation is required for adaptive thermogenesis as a result of direct actions on gene regulation and indirectly by stimulation of D2 activity. In BAT, adrenergic signaling through the *β*3-adrenergic receptor (*β*3-AR) stimulates UCP1 gene expression through the activation of protein kinase A and D2 via deubiquitination and promotes thermogenesis and weight loss. In this metabolic process, FT3 is also required to induce UCP1 gene upregulation [11].

There are several types of iodothyronine deiodinases, including two activating enzymes (D1 and D2) and one inactivating enzyme (D3). D1 is expressed at high levels in the liver, kidney, and thyroid; D2 in the brain, pituitary, thyroid, and BAT; and D3 in the skin and vascular tissue. D2 is the primary enzyme responsible for the rapid increases in intracellular T3 in specific tissues as well as the primary producer of serum T3 in humans [4]. The D2 enzyme has a short half-life due to ubiquitination and subsequent proteasome degradation [12, 13]. Deubiquitination, which increases D2 activity, is stimulated by adrenergic activation or by low levels of serum T4 [14, 15]. D2 is expressed in key TH-responsive tissues, such as the brain, skeletal muscle, and BAT. FT3 is preserved in these tissues as serum FT4 levels decrease. The FT3 generated intracellularly by D2 is transferred to the nucleus for the regulation of gene transcription. D2 activity is critical for the synergism of TH and signaling for the regulation of thermogenesis in BAT [16]. Forty years ago, Doniach proposed a possible mechanism for the stimulation of thermogenesis in BAT by TSH [6]. Furthermore, a more recent mouse study demonstrated that D2 was essential for thyroid-sympathetic synergism required for thermal homeostasis in BAT, and the deletion of the TSH receptor resulted in impaired BAT thermogenesis [5]. In an FDG-PET imaging study of mice, Jeanguillaume et al. confirmed that increased glucose uptake in fat is, generally, an indication of the presence of UPC1-positive BAT [3].

TSH increases basal and FT3-stimulated UCP1 and Dio2 expression as well as D2 activity. TSH can increase basal UCP1 levels, which is synergized by the effect of T3 on UCP1 [17]. In this study, the authors also proposed TSH activity as an alternative pathway in thermogenesis regulation by BAT. Endo and Kobayashi reported that TSH was involved in the regulation of UCP1 expression and thermogenesis in BAT to protect against a further decrease in body temperature in a mouse hypothyroid model [7].

Although several reports have discussed the correlation between serum TH levels and the visualization of BAT on FDG-PET or PET/CT, there is no definite consensus on this association. Lahesmaa et al. evaluated 10 patients with hyperthyroidism and healthy and euthyroid individuals by FDG-PET and found greater glucose uptake in BAT of hyperthyroid patients than that of controls [8]. On the other hand, a recently published controversial report claims that nine of 10 study subjects with Graves' disease had no detectable active BAT on FDG-PET/CT [18]. This group also reported significantly lower FT3 and FT4 levels in 75 BAT-positive subjects than in BAT-negative subjects and proposed the lower levels of TH needed for higher BAT thermogenesis as a possible cause. Furthermore, there is a recent case report about BAT visualization on FDG-PET/CT in severe primary hypothyroidism that suggested a potential role of TSH and TRH as regulators of BAT activation [19].

In radioiodine therapy (RIT) for metastasis after total thyroidectomy for differentiated thyroid carcinoma, patients were instructed to adhere to a low-iodine diet and discontinue TH supplementation for at least 2-3 weeks before RIT so that the patients would tend to exhibit temporary hypothyroidism (low FT3 and FT4 and elevated TSH). In thyroidal ablation therapy after total thyroidectomy for differentiated thyroid carcinoma, patients were instructed to adhere to a low-iodine diet and received injections of rhTSH 1 and 2 days before RIT instead of TH withdrawal to temporally elevate serum TSH. FT3 and FT4 levels tended to be within the normal range in most patients who underwent thyroidal ablation because of continuous TH replacement, as in this study. Our results showed that temporarily or continuously elevated TSH did not have a significant influence on BAT visualization on whole-body FDG-PET/CT imaging. In addition, we found that suppression of serum TSH in the hyperthyroidism group had no significant effect on the visualization of activated BAT on FDG-PET/CT imaging. Only temperature either on the day on or the day before FDG-PET/CT imaging was a significant parameter impacting the visualization of BAT, as described before [20–24].

In the current study, the small number of subjects and the lack of a cooling protocol to stimulate BAT group were major study limitations. Since this study was a retrospective one, it was not easy to register enrollees who confirmed serum TSH, FT3, and FT4 in the BAT group. Further studies with prospective design and basic research might be needed to clarify the association between BAT visualization and thyroid hormones.

In summary, we investigated the influence of TH on the visualization of activated BAT on FDG-PET/CT images of patients with various conditions. Although TH was considered to be associated with thermogenesis through UCP1 gene expression and D2 in BAT, elevated TSH status before RIT, hyperthyroidism, or hypothyroidism did not significantly impact BAT visualization and the interpretation of clinical FDG-PET/CT images.

## Figures and Tables

**Table 1:** Patient characteristics and summary of each group.

Group	THW	rhTSH	Hypothyroidism	Hyperthyroidism	BAT	*p* value
*N*	21	8	9	14	10	
BAT uptake	0	0	0	0	10	
Age (y-o)	61.6 ± 14.9	48.4 ± 14.1	65.8 ± 17.5	50.0 ± 13.3	52.5 ± 22.9	n.s.
Gender (M : F)	7 : 14	4 : 4	4 : 5	6 : 8	2 : 8	n.s.
Body weight (kg)	62.7 ± 14.6	66.6 ± 18.7	50.7 ± 12.6	60.0 ± 18.0	50.1 ± 9.4	n.s.
TSH (*μ*IU/mL)	50.79 ± 34.54	191.80 ± 73.97	23.02 ± 27.77	0.03 ± 0.04	2.58 ± 2.72	<0.0001
FT3 (pg/mL)	1.54 ± 0.27	2.85 ± 0.33	1.99 ± 0.39	7.11 ± 7.92	2.74 ± 0.11	<0.0001
FT4 (ng/dL)	0.48 ± 0.08	1.29 ± 0.12	0.87 ± 0.28	1.89 ± 1.17	1.22 ± 0.03	<0.0001
T-Cho (mg/dL)	244 ± 44	165 ± 26	156 ± 46	146 ± 41	203 ± 28	<0.0001
Min. temp. of the day (°C)	14.2 ± 8.0	18.0 ± 5.5	19.9 ± 6.3	14.9 ± 8.2	5.1 ± 7.1	<0.01
Max. temp. of the day (°C)	23.0 ± 6.2	25.1 ± 5.0	28.4 ± 4.2	21.9 ± 6.2	13.8 ± 5.5	<0.01
Max. temp. of 1 day before (°C)	23.2 ± 6.3	24.9 ± 3.8	28.6 ± 3.6	21.6 ± 7.6	13.9 ± 4.4	<0.01
Difference temp. (°C)	9.0 ± 3.4	6.9 ± 3.9	8.7 ± 3.4	6.8 ± 4.2	8.8 ± 1.1	n.s.

Data: expressed as mean ± s.d.

**Table 2:** Visualization of BAT on ^18^F-FDG-PET/CT and related parameters.

#	Gender	Age	Body weight (kg)	Brown fat uptake	TSH (*μ*IU/mL)	FT3 (pg/mL)	FT4 (ng/dL)	T-Cho (mg/dL)	min. temp. of the day (°C)	max. temp. of the day before (°C)	Difference of temp. (°C)^*∗*^
THW											
(1)	F	29	65.0	negative	77.67	1.26	0.40	237	25	30	5
(2)	F	32	63.9	negative	20.44	1.51	0.40	255	14	19	5
(3)	F	42	48.7	negative	93.51	1.78	0.53	-	17	26	9
(4)	F	49	54.0	negative	124.48	1.60	0.46	287	12	22	10
(5)	M	50	84.0	negative	24.42	1.53	0.52	-	25	31	6
(6)	F	55	91.0	negative	13.43	2.06	0.54	296	12	20	8
(7)	F	56	52.0	negative	67.10	1.69	0.42	239	2	12	10
(8)	F	56	88.7	negative	18.92	1.67	0.46	-	20	26	6
(9)	M	64	64.0	negative	56.75	2.04	0.48	265	12	21	9
(10)	M	65	70.2	negative	64.25	1.64	0.47	175	22	29	7
(11)	F	65	51.6	negative	54.47	1.36	0.44	251	15	25	10
(12)	M	66	67.2	negative	122.54	1.00	0.40	207	−1	18	19
(13)	F	68	57.2	negative	72.49	1.40	0.40	342	14	21	7
(14)	M	68	57.6	negative	8.01	1.63	0.57	-	6	14	8
(15)	F	69	37.7	negative	19.94	1.35	0.56	287	22	34	12
(16)	F	70	47.6	negative	35.78	1.06	0.51	-	3	19	16
(17)	F	72	49.0	negative	58.63	1.58	0.40	225	18	27	9
(18)	M	75	84.2	negative	25.74	1.65	0.65	209	21	28	7
(19)	F	77	68.0	negative	35.08	1.34	0.41	271	25	32	7
(20)	F	78	51.4	negative	10.13	1.44	0.67	200	12	19	7
(21)	M	87	63.7	negative	62.88	1.71	0.49	204	3	14	11

rhTSH											
(22)	F	20	53.9	negative	151.24	2.44	1.21	217	15	19	4
(23)	M	41	100.4	negative	87.31	3.20	1.17	147	14	25	11
(24)	M	45	51.8	negative	175.22	2.55	1.48	165	20	27	7
(25)	M	47	81.0	negative	158.08	2.85	1.15	155	21	23	2
(26)	F	50	67.0	negative	149.42	3.31	1.27	175	11	24	13
(27)	F	59	78.4	negative	223.90	2.67	1.45	161	25	28	3
(28)	F	60	45.6	negative	281.10	3.14	1.26	217	13	22	9
(29)	M	65	54.8	negative	308.10	2.67	1.34	139	25	31	6

Hypothyroidism											
(30)	M	25	43.0	negative	24.83	2.21	0.98	-	23	31	8
(31)	F	56	71.7	negative	5.56	2.11	1.18	256	23	28	5
(32)	M	60	46.1	negative	9.88	2.17	0.94	193	23	30	7
(33)	F	70	45.9	negative	5.26	2.35	0.96	184	6	23	17
(34)	F	70	46.0	negative	3.78	1.56	1.12	136	24	31	7
(35)	M	73	58.8	negative	77.99	1.14	0.40	132	14	23	9
(36)	F	77	37.8	negative	5.07	2.15	0.87	127	21	29	8
(37)	F	78	38.2	negative	12.41	1.98	1.00	131	26	34	8
(38)	M	83	68.7	negative	62.36	2.26	0.40	121	19	28	9

Hyperthyroidism											
(39)	F	32	46.2	negative	0.01	3.22	1.93	120	6	16	10
(40)	M	33	63.6	negative	0.01	4.76	1.67	124	26	30	4
(41)	M	38	49.1	negative	0.00	30.00	4.89	161	19	29	10
(42)	M	38	102.5	negative	0.07	4.59	0.98	-	−1	9	10
(43)	F	39	44.0	negative	0.01	3.33	1.02	-	19	22	3
(44)	F	51	62.4	negative	0.05	3.98	1.74	141	16	20	4
(45)	F	52	83.0	negative	0.01	8.42	2.11	149	3	14	11
(46)	F	52	37.9	negative	0.01	4.41	0.95	94	17	30	13
(47)	F	52	51.2	negative	0.01	3.59	1.42	205	17	28	11
(48)	F	53	81.5	negative	0.01	3.90	1.17	-	25	28	3
(49)	M	54	58.5	negative	0.01	19.72	3.99	107	11	19	8
(50)	M	62	57.0	negative	0.13	2.32	1.34	-	18	18	0
(51)	M	64	59.8	negative	0.01	4.90	2.12	121	9	10	1
(52)	F	80	43.4	negative	0.01	2.38	1.11	210	23	30	7

BAT											
(53)	F	24	60.6	positive	3.58	2.49	1.07	-	3	13	10
(54)	F	27	35.2	positive	0.48	2.94	1.29	-	0	12	12
(55)	M	27	67.0	positive	1.16	3.55	1.13	211	21	23	2
(56)	F	41	49.7	positive	0.49	3.19	1.25	244	2	9	7
(57)	F	50	50.0	positive	4.88	2.35	1.32	200	6	17	11
(58)	F	50	36.6	positive	1.13	2.45	1.31	168	9	15	6
(59)	F	70	51.8	positive	9.19	2.76	1.30	212	12	18	6
(60)	M	71	50.2	positive	2.55	3.15	0.96	-	−1	11	12
(61)	F	82	50.0	positive	1.16	2.36	1.36	177	0	10	10
(62)	F	83	50.0	positive	1.15	2.12	1.25	175	−1	11	12

-: not determined. ^*∗*^Difference of temperature: (max. temp. of 1 day before) − (min. temp. of the day) (°C); normal range of TSH: 0.35–4.94 *μ*IU/mL, FT3: 1.71–3.71 pg/mL, and FT4: 0.70–1.48 ng/dL, T-Cho: 129–238 mg/dL.

**Table 3:** Comparison of parameters between BAT negative and BAT positive in this study population.

	BAT negative	BAT positive	*p* value
*N*	52	10	
Age (y-o)	57.2 ± 16.0	52.5 ± 22.9	n.s.
Gender (M : F)	21 : 31	2 : 8	n.s.
Body weight (kg)	60.5 ± 16.3	50.1 ± 9.4	n.s.
TSH (*μ*IU/mL)	54.00 ± 72.74	2.58 ± 2.73	n.s.
FT3 (pg/mL)	3.32 ± 4.65	2.74 ± 0.46	n.s.
FT4 (ng/dL)	1.05 ± 0.84	1.22 ± 0.13	n.s.
T-Cho (mg/dL)	189 ± 59	203 ± 28	n.s.
Min. temp. of the day (°C)	16.0 ± 7.6	5.1 ± 7.1	<0.01
Max. temp. of the day (°C)	24.0 ± 6.1	13.8 ± 5.5	<0.01
Max. temp. of 1 day before (°C)	23.7 ± 6.5	13.9 ± 4.4	<0.01
Difference temp. (°C)	8.0 ± 3.8	8.8 ± 3.4	n.s.

Data: expressed as mean ± s.d.; if *p* value is less than 0.01, it is as significant; Wilcoxon rank-sum *χ*^2^-square test.

## Supplementary Materials

Supplementary 1 Whole-body maximum intensity projection images of FDG-PET/CT of the hyperthyroidism group. Each image corresponds to patients #(39)–#(52) of Table 2.

Supplementary 2 Whole-body maximum intensity projection images of FDG-PET/CT of the BAT group. Each image corresponds to patients #(53)–#(62) of Table 2.
